# Active Microbiome Structure and Functional Analyses of Freshwater Benthic Biofilm Samples Influenced by RNA Extraction Methods

**DOI:** 10.3389/fmicb.2021.588025

**Published:** 2021-04-16

**Authors:** Yuan Yao, Subramanya Rao, Olivier Habimana

**Affiliations:** ^1^The School of Biological Sciences, Faculty of Science, The University of Hong Kong, Pokfulam, Hong Kong; ^2^The University of Hong Kong Shenzhen Institute of Research and Innovation (HKU-SIRI), Shenzhen, China

**Keywords:** RNA extraction, benthic biofilms, freshwater ecosystems, method comparison, 16S rRNA gene sequencing, meta-transcriptomics

## Abstract

Advances in high-throughput sequencing technologies have enabled extensive studies of freshwater biofilms and significant breakthroughs in biofilm meta-omics. To date, however, no standardized protocols have been developed for the effective isolation of RNA from freshwater benthic biofilms. In this study, we compared column-based kit RNA extraction with five RNAzol-based extractions, differentiated by various protocol modifications. The RNA products were then evaluated to determine their integrity, purity and yield and were subjected to meta-transcriptomic sequencing and analysis. Significant discrepancies in the relative abundance of active communities and structures of eukaryotic, bacterial, archaebacterial, and viral communities were observed as direct outcomes of the tested RNA extraction methods. The column isolation-based group was characterized by the highest relative abundance of Archaea and Eukaryota, while the organic isolation-based groups commonly had the highest relative abundances of Prokaryota (bacteria). Kit extraction methods provided the best outcomes in terms of high-quality RNA yield and integrity. However, these methods were deemed questionable for studies of active bacterial communities and may contribute a significant degree of bias to the interpretation of downstream meta-transcriptomic analyses.

## Introduction

In freshwater environments, benthic biofilms can be considered “cities of microorganisms” that attach to abiotic surfaces and embed into extracellular polymeric substances. These freshwater biofilms have been identified as major sites of carbon and nutrient cycling (Lear et al., 2012) and are thought to play an important role in the maintenance and monitoring of freshwater health. With assistance from advanced meta-omics tools, ongoing environmental biofilm research has led to significant breakthroughs in the study of structures, functional attributes, and metabolism in microbial communities (Rani and Babu, 2018). Only recently, insights into the phenomes of freshwater microbial communities were gained through the integration of information gained using different meta-omics approaches (Beale et al., 2017; Pu et al., 2019).

The study of biofilm community structures and their functional attributes require a proper nucleic acid extraction, regardless of the sequencing platform or molecular analysis method. Indeed, reliable high-quality total nucleic acid (RNA) extraction is a prerequisite for recovering messenger RNA (mRNA) for gene expression studies based on next-generation sequencing, quantitative reverse transcription polymerase chain reaction (qRT-PCR) or microarrays, as low-quality total RNA may strongly compromise the experimental results and waste time, labor, and resources (Fleige and Pfaffl, 2006; Riedmaier et al., 2010). In essence, the recovery of mRNA, needed for expression studies and transcripomic analyses may be influenced by the initial method used for extracting high quality total RNA. A plethora of nucleic acid extraction methods exist, ranging from commercial kits to protocols involving homemade reagents. These methods include diverse mechanical, chemical, and/or enzymatic lysis methods and diverse post-extraction purification methods. In one example, Chang and colleagues developed “in-house” methods for soil microbiome studies (Chang et al., 1993), while other researchers have used the traditional phenol-chloroform method (Fleming et al., 1998) for isolating ribosomal RNA to analyze bacterial communities and their functional profiles (Sessitsch et al., 2002). Although several methods to extract total RNA from environmental samples have been described in the literature (Hurt et al., 2001; Sessitsch et al., 2002; Wang et al., 2012), few published studies have tested and compared these methods when applied to environmental biofilm samples.

It is difficult to obtain high-quality total RNA from environmental biofilm samples, as these molecules are easily degraded by intracellular or extracellular RNases even before sampling and extraction. Accordingly, the ideal method of extracting high-quality total RNA from soil and sediment samples has been debated over the last three decades (Hurt et al., 2001; Sessitsch et al., 2002; Wang et al., 2012). Contamination by humic substances, which is difficult to remove using phenol extraction methods, represents one of the main challenges associated with high-quality total RNA isolation from soil, although the low yield and adsorption of total RNA by soil are also important challenges (Wang et al., 2012). Although various specialized and standardized kits have been developed and made commercially available for the industrial extraction of total RNA from a range of sample types (e.g., soil, feces, sediment), total RNA recovery from environmental biofilm samples remains enigmatic, especially with regard to the relative effectiveness of extraction methods. This is particularly true when comparing the use of traditional total RNA extraction methods that maximize the yield from low-biomass biofilm samples with the use of kit extraction methods that focus on higher integrity at the expense of total RNA yield (Jahn et al., 2008).

As indicated, the high-quality and high-yield extraction of total RNA from environmental biofilms is a highly important requisite to downstream applications. However, different extraction methods may introduce variability in the composition of recovered mRNA and subsequently yield a biased sequencing result. However, very few studies have compared the effects of different total RNA extraction methods on environmental biofilm samples, and their respective recovered mRNA in terms of subsequent gene expression profile obtained via meta-transcriptome sequencing. The total RNA extraction strategies applied in this study ranged from a standardized column-based kit isolation, to five organic-based extraction methods, generating a total of six extraction groups. Hence, the objectives of this study were to (1) examine the purity, yield and integrity of total RNA extracts following six different extraction procedures; (2) assess and compare the active microbiomes associated with benthic freshwater biofilms using recovered mRNA from previously extracted total RNA from each extraction procedures; (3) identify the functional profiles of the active microbiomes resulting outcomes from each extraction procedure, and (4) analyze and compare the differential gene expression profile outcomes from the six extraction procedures performed in this study.

## Materials and Methods

### Study Design

**FIGURE 1:**
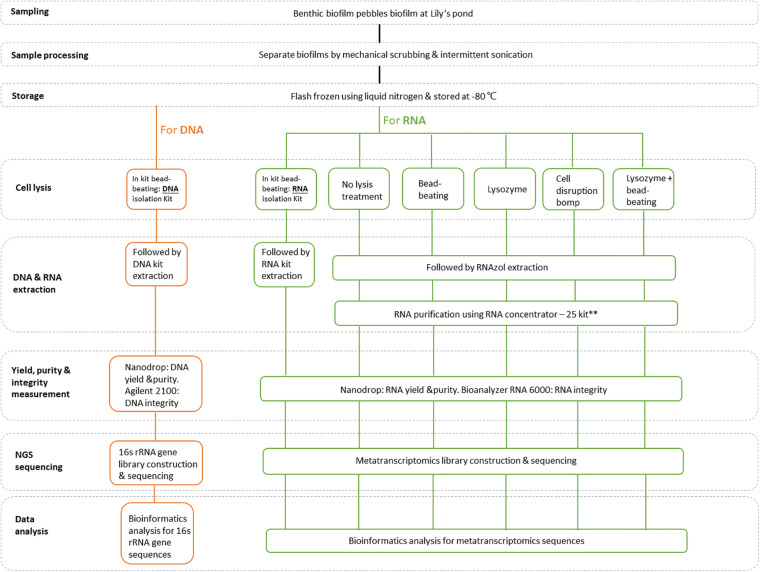
A schematic overview of the study design. **Since the total RNA samples obtained using non-column-based extraction procedures were initially of poor quality, an additional purification step was performed on half of the sample using an RNA Clean & Concentrator-25 kit (Zymo Research, United States).

A schematic diagram of the study design is presented in Figure 1. Biofilms from freshwater pebbles were used in this study. Following collection, biofilm samples were separated from pebbles by mechanical scrubbing and intermittent sonication. Biofilm samples were then quickly frozen using liquid nitrogen and stored at −80°C for further nucleic acid extraction. Genomic DNA (gDNA) was extracted from stored biofilm samples using a column-based method, following assessment of purity, yield and integrity, before 16S rRNA sequencing and species annotation analysis. Total RNA extraction of biofilm samples was performed using six extraction methods categorized in six different groups. One standardized group was generated by following a (i) column isolation-based method (Quick-RNA Fecal/Soil Microbe Microprep kit: Zymo Research, R2040). The other five extraction groups were subjected to organic isolation-based methods: (ii) RNAzol^®^ (RN190; Molecular Research Center, United States) extraction with different pretreatment cell disruption steps, (iii) including no cell disruption (RNAzol only group), (iv) bead-beating, (v) lysozyme, and (vi) lysozyme + bead-beating. Since the organic isolation-based groups yielded RNA with relatively low purity after initial extraction, half of the non-column-based extracted RNA samples were subjected to an additional purification step using an RNA Concentrator-25^TM^ kit (Zymo Research, United States). All total RNA extracts were assessed for purity, yield and integrity, and pooled for subsequent mRNA recovery prior to metatranscriptome sequencing and bioinformatics analysis.

### Biofilm Sampling, Processing and Storage

Submerged pebbles covered with benthic biofilms were collected from the lily pond at the Main Building of The University of Hong Kong (Pok Fu Lam Rd, Lung Fu Shan). In total, we conducted four independent sampling procedures on different dates (Cf. Supplementary Table 1). For each independent sampling procedure, duplicates for each DNA and RNA extraction group were created. On each sampling day, an additional biofilm sample were collected for conducting independent dry weight measurements of the biomass. For each trial, 8–10 pebbles with size ranging from 30 to 60 mm were collected in jars (max volume: 1.2 L) and later submerged in a volume of bulk limnetic pond water. The volume of pond water was sufficient to cover all pebbles, and the final volume of mixture of pebbels and pond water was 1 L. The biofilms were separated from the pebbles by gentle mechanical scrubbing and intermittent sonication (15-s on/15-s off cycle) for 7 min and 28 kHz frequency (Kan-Pacific, Hong Kong) (Pu et al., 2019). The sampled biofilms were resuspended in ultrapure water and centrifuged at 2,100 × *g* for 10 min. The supernatants were gently removed without disturbing the biofilm pellets. The same biofilm sample was then homogenized by up and down pipetting and subsequently 400-μl aliquots of this biofilm biomass suspension was then placed in separate Eppendorf tubes. The biofilm aliquots were then flash-frozen using liquid nitrogen and stored at −80°C. One aliquot of each independent repeat was freeze-dried to obtain a dry weight measurement. The procedure for freeze drying is described as follows: the biofilm aliquot in Eppendorf tube was covered with punctured parafilm to allow water evaporation. The biofilm aliquot was then placed in a lid-less 50 ml Falcon tube, which was also placed in the freeze-dryer’s sample holder flask. The freeze dryer was adjusted to temperatures below −30°C and pressure below 0.5 mbar. The biofilm sample was dried overnight and the final product was fully lyophilized. The benthic biofilm sampling metadata, including the ambient temperature, water temperature, water pH, sampling depth, and sampling time, are provided in Supplementary Table 1.

### Genomic DNA Isolation, Sequencing and Data Analysis

To obtain an overall understanding of the microbial communities structure and active microbiome profile, it is important to employ a multiphasic approach. Here, the identification of microbial community structures utilizing genomic DNA extraction and 16S rRNA sequencing approaches were employed. Genomic DNA (gDNA) isolation from the above-described benthic biofilms and the subsequent sequencing and analysis protocols were performed as recently described (Pu et al., 2019), with slight modifications. The purity and yield of the extracted DNA were assessed using a BioDrop device (Biodrop Ltd., United Kingdom), and the integrity was determined using an Agilent 2100 (Agilent Technologies, United States). Extracted DNA from four independent sampling procedures yielding eight replicates were pooled and sent to Novogene for amplicon sequencing and analysis. The 16S rRNA V3–V4 region was amplified in each sample, using the bacterial primers 341F: CCTAYGGGRBGCASCAG and 806R: GGACTACNNGGGTATCTAAT with a unique barcode (TGACCA, ACTGAT) (Zakrzewski et al., 2012). Phusion High-Fidelity PCR Master Mix (New England Biolabs, United States) was used for all of the PCR reactions. PCR products were quantified by running a 2% gel electrophoresis, and samples with bright main strip between 400 and 450 bp were chosen for purification with Qiagen Gel Extraction Kit (Qiagen, Germany). Next, sequencing libraries were generated using the TruSeq DNA PCR-free sample preparation kit (Illumina, United States) according to the manufacturer’s instructions. The library was subjected to a quality control analysis using a Qubit 2.0 Fluorometer (Thermo Scientific, United States) and subsequently sequenced on the Illumina HiSeq2500 platform (Illumina Inc., United States) to generate 250-bp paired-end reads.

Following a cleanup step comprising the truncation of barcode and primer sequences, the paired-end reads were merged using FLASH (Magoc and Salzberg, 2011) to obtain raw reads that were later subjected to quality filtering to obtain high-quality clean reads (Bokulich et al., 2013) according to the QIIME quality control process (Caporaso et al., 2010b). To obtain effective tags, the UCHIME algorithm was used to compare the cleaned read sequences with a reference Gold database (Edgar et al., 2011) and thus enable the removal of chimera sequences. Uparse (Edgar, 2013) was used to conduct the sequence analysis, and sequences with ≥97% similarity were assigned to the same operational taxonomic unit (OTU). A representative sequence from each OTU was then screened for species annotation using the GreenGene Database (DeSantis et al., 2006) based on the RDP Classifier (Wang et al., 2007). The phylogenetic relationships of different OTUs, differences between the dominant species in the samples (groups) and multiple sequence alignments were analyzed against the “Core Set” dataset in the GreenGene database using PyNAST v1.2 (Caporaso et al., 2010a).

### RNA Extraction

For each extraction method, eight replicates were generated from four independent extractions to yield a total of 48 extracted RNA samples. All RNA samples were analyzed using a BioDrop Touch Duo device (No. 80-3006-61) to determine the purity and yield. A Bioanalyzer RNA 6000 device was used to assess RNA integrity. However, given the resulting poor RNA yield observed for non-column-based RNA extraction strategies (chemical-based extraction groups) employed in this study, an extra purification step using RNA Clean & Concentrator-25 kit (Zymo Research, United States) was performed on half of the non-column-based RNA extracts. Hence, RNA extracts for each extraction strategy were pooled and then further sent for library construction and metatranscriptomic sequencing and analysis (*n*, indicating the number of pooled RNA extracts): RNA from column-based kit extraction (*n* = 8);

RNA from RNAzol Only extraction (*n* = 4); RNA from beads beating extraction (*n* = 4); RNA from lysozyme extraction (*n* = 4); RNA from cell disruption extraction (*n* = 4); RNA from lysozyme + beads beating extractions (*n* = 4). For metatranscriptomics sequencing and analysis, there were no replicates for each extraction group as they have been all pooled prior to this step to obtain sufficient yield.

Regarding the six RNA extraction procedures, the following strategies were performed to assess the effects of different RNA extraction methods on downstream meta-transcriptomic experiments. Tests consisting of various modifications and pretreatments of the RNAzol protocol are described from (2.1) to (2.5) here below.

#### Column-Based Kit Isolation (K)

The Quick-RNA Fecal/Soil Microbe Microprep kit (Zymo Research, R2040) was used according to the kit protocol, which included a cell disruption step with 0.1- and 0.5- mm beads.

#### RNAzol Only Group (R)

The RNAzol protocol was followed based on manufacturer’s instructions. RNAzol acts as a chemical lysis reagent to disrupt the outer membranes of bacterial cells.

#### Bead-Beating Group (B)

The cells were first disrupted by two alternate cycles of 40 s vortexing and 20 s rest using 0.150 to 0.212 mm glass beads (Sigma, G1145-100G), followed by standard RNAzol extraction.

#### Lysozyme Group (L)

A 60-μl volume of lysozyme (5 mg/ml) (Sigma, L6876-1G) was added to the liquid biofilm sample, together with 30 μl of NaCl-EDTA (0.3 M, 0.02 M) as the activating buffer. The sample was then vortexed for 30 s, incubated in a 37°C water bath for 2 min, and vortexed for 30 s prior to standard RNAzol extraction.

#### Cell Disruption Bomb (P)

To set up this equipment, a pressure regulator (SMT Detendeur, DL230 – DI230) was connected to a nitrogen cylinder (Linde Nitrogen, UN 1066). This procedure comprised slow compression and fast depression (Hemmingsen and Hemmingsen, 1980). In the slow compression stage, the cell disruption bomb was filled stepwise with nitrogen gas up to 1,500 psi in 10 steps of 150 psi each, followed by an 8-min standby period. The slow compression period enabled the bacterial cells to adapt gradually to the increased pressure. Once the cells had adapted to a pressure of 1,500 psi, the bomb was subjected to fast decompression to release all pressure within 15 s. This sudden decrease in pressure disrupted the bacterial walls. Subsequently, RNA was extracted using the RNAzol method.

#### Lysozyme + Bead-Beating Group (LB)

This method combined the approaches of lysozyme and bead-beating for cell disruption, followed by RNAzol extraction. All of the extracted RNA samples were stored at −80°C.

### Total RNA Sample Quality Control

Total RNA samples were subjected to three quality control analyses prior to library construction: (A) NanoDrop analysis (Thermo Fisher, United States) to assess RNA purity (OD_260_/OD_280__;_ OD_260_/OD_230_); (B) Qubit analysis (Thermo Fisher, United States) to determine the RNA yield; and (C) Agilent 2100 Bio-analyzer analysis (Agilent Technologies) to verify RNA integrity. The significance of the results was determined using analysis of variance (ANOVA) and SPSS Statistics software, version 24.0 (IBM, United States).

### Metatranscriptome Sequencing and Assembly

After the RNA sample quality assessment, the extracted total RNA was first subjected to ribosomal RNA removal using the Ribo-Zero rRNA Removal Kit (Illumina, Inc.). The mRNA was fragmented randomly before cDNA synthesis, which were achived using the NEBNext Ultra^TM^ II Directional RNA Library Prep Kit for Illumina (Illumina, Inc.). The final cDNA library preparation involved a multistep process that included purification, terminal repair, A-tailing, sequencing adapter ligation, size selection and PCR enrichment. For library QC, the library concentration was quantified using a Qubit 2.0 fluorometer and adjusted to 2 ng/μl. Next, the insert size was checked using an Agilent 2100 Bio-analyzer and quantified to a greater accuracy by quantitative PCR. The PCR products were subjected to standard quantification and purification procedures (Novogene Bioinformatics Technology). Finally, the libraries were sequenced on an Illumina HiSeq2500 platform to generate 250-bp paired-end reads. The Illumina 16S rRNA gene amplicon sequencing and metatranscriptome reads were deposited in the NCBI-Sequence Read Archive (SRA) under the following accession reference number (PRJNA594486).

The RNA-seq reads were processed routinely using the FastQC program to eliminate adapter contamination and low-quality reads (low quality nucleotides constitute ≥50% of the read) (Beg et al., 2000), as well as reads when uncertain nucleotides constitute more than 10% of the read (*n* ≥ 10%). To further eliminating tRNA and rRNA, the remaining reads were subjected to BlastN with a minimum alignment bit score of 54, using a filtering database that comprised the complete rRNA loci and tRNA sequences of bacteria, archaea, and eukaryotes from the National Center for Biotechnology (NCBI) and SILVA databases (Pruesse et al., 2007). In total, 76.95 GB clean bases were generated for all groups. The remaining mRNA sequences that passed this rRNA/tRNA filtering process were assembled using Trinity (version r20140413pl). All of the samples were then integrated, and redundant samples were removed using CD-HIT-EST (identity threshold set to 0.95) to obtain unigenes.

### Taxonomic Annotation

Unigenes with Bacteria, Fungi, Archae, and Viruses were aligned with the NR database of the NCBI (*e*-value ≤ 1e^–5^) using DIAMOND software (BlastP), while allowing for the acquisition of taxonomic ranks. According to a previous study, minimum bit score thresholds of 148, 110, and 74 can be used, respectively, for phylogenetic and functional assignments at the genus level (>80% confidence level), phylogenetic and functional assignments at the family level (>80% confidence level) and reliable function (COG) assignments (>95% confidence level) (Leimena et al., 2013). Phylum-level phylogenetic profiles were based on mRNA reads with minimum bit alignment scores of 148, and the LCA algorithm (applied in MEGAN software system) was used to select the highest rank for species annotation and to ensure the biological significance (Huson et al., 2011). The top 35 phyla in each sample were selected from the species annotation results and relative abundance data and were clustered using their taxonomy information and the inter-sample differences to generate a heat-map of phylum relative abundance.

### KEGG Functional Annotation, eggNOG and CAZy Analyses

To elucidate the microbial structures and functions in the biofilms, a Kyoto Encyclopedia of Genes and Genomes (KEGG) functional annotation analysis, homologous gene cluster analysis (eggNOG) and carbohydrate enzyme analysis (CAZy) were performed by mapping functionally annotated unigenes to different functional protein databases using BLAST software. As each mapping unigene yielded more than one outcome, BLAST Coverage Ratios (BCRs) of the reference and query genes were calculated to establish a BCR (Ref) and BCR (Que) > 40%, and these values were used to ensure biological significance. Once predicted, the unigenes were assigned to COGs (Tatusov et al., 2000) via blast searches against the COG database using the NCBI (Berbee, 2001). An *e*-value < 10^–6^ was used for the COG assignments. The KEGG functional annotation analysis was conducted using the KEGG Automatic Annotation Server (Moriya et al., 2007; Berendsen et al., 2012) and a bidirectional best hit method was used to assign the identified proteins.

### Differential Gene Expression Analysis

An expectation-maximization (RSEM) (Li and Dewey, 2011) routine was used to align the unigenes with RNA-Seq data. The number of unigene reads mapped to each biofilm extraction method strategy was then derived. The relative gene expression was determined by counting the number of unigenes assigned to a particular protein-encoding gene. Subsequently, the data were normalized by dividing each gene count by the total mRNA read count of each dataset and multiplying this value by the average total mRNA read count across all datasets (Dillies et al., 2013). The transcripts were calculated and normalized to fragments per kilobase of transcript sequence per million mapped reads (FPKM) to represent the gene expression levels (Trapnell et al., 2010). DESeq software was used to identify differentially expressed genes (DEGs) via pair-wise comparisons, and genes with a corrected padj < 0.05 and an absolute log2 fold-change value ≥1 were considered differentially expressed. In these comparisons, column-based kit isolation group was employed as control group, and chemical-based extractions were considered as treatment groups. Differential gene cluster analysis was used to determine the patterns that emerged under different experimental conditions. Each challenge–control combination produced a differential gene set, and the FPKM values of all the combined differential gene sets in each experimental group/sample were included in the cluster analysis.

Quantitative metabolic mapping of the metatranscriptome profiles was performed by mapping the KEGG annotations of identified protein sequences into metabolic pathway maps, using the iPath v2 module (Bokulich et al., 2013). In these maps, the gene expression associated with a metabolic pathway was indicated by the line width, which was determined using the log2 read count values of KEGG-annotated proteins. Reads with alignment bit-scores ≥74 were used to create global metabolic activity pathways.

### Data Availability

The data that support the findings of this study are available from the corresponding author upon reasonable request.

## Results

### Distribution of Bacterial Communities Within Freshwater Benthic Biofilms Based on a 16S rRNA Amplicon Sequencing Analysis

First, the bacterial community profiles in freshwater benthic biofilms were assessed to provide a reference for this study. Biofilm DNA was subjected to an amplicon-based sequencing approach that targeted the 16S rRNA V3-V4 region. The generated 16S metagenomics sequence data from the sampled benthic biofilms contained 98,675 raw tags, 97,233 clean tags and 92,460 effective tags (after chimera removal), with an average read length of 250 nucleotides (Supplementary Table 2). Cyanobacteria and Proteobacteria were the most abundant phyla in the benthic biofilms, with relative abundances of 40 and 35%, respectively (Supplementary Figure 1). Bacteroidetes, Firmicutes, Nitrospirae, and Chloroflexi were less abundant in the sampled freshwater pebble-benthic biofilms, with relative abundances of 4.6, 4.5, 4.1, and 2.7%, respectively.

### Purity, Integrity and Yield Assessments of the Extracted RNA

It was noticed that the quantity of RNA extracts of each replicate from pebbles biofilms was too low for sequencing, hence, for each extraction method, we pooled the replicates and sent these for further library construction. The dry weight biomass from four independently sampled biofilms was recorded as 0.0635, 0.0151, 0.0253, and 0.0353 g, respectively. RNA extractions from the standardized column isolation-based approach yielded the best results in terms of yield and quality compared with the other tested RNA extraction methods. The column isolation-based group yielded a significantly higher RNA integrity level (*p* < 0.05, one-way ANOVA), as well as a non-significantly higher purity level and yield. Obtained values of RNA purity (A_260__/__280_; A_260__/__230_), yield (μg/g dry sample) and integrity values from column-based extractions were 2.07 ± 0.03, 2.13 ± 0.36, 298 ± 136, and 5.63 ± 0.48 (Table 1A), respectively. Similar yield outcomes were observed when comparing the five chemically based extraction methods, irrespective of whether a purification step was implemented prior to, or after RNA extractions. For chemically based extractions conducted prior to a purification step, averaged values of RNA purity (A_260__/__280_; A_260__/__230_), yield (μg/g dry sample) were found at 1.62 ± 0.04, 0.83 ± 0.11, and 302.15 ± 106.43, respectively (Table 1B). Obtained values of RNA purity (A_260__/__280_; A_260__/__230_), yield (μg/g dry sample) and integrity values for the chemically based extractions following a purification step averaged at 1.92 ± 0.13, 1.54 ± 0.31, 205.16 ± 110.28, and 2.63 ± 0.11, respectively (Table 1C). Increased RNA purity values were generally observed following the addition of a RNA purification step after conducting chemically based extractions (*p* < 0.05, paired sample *t* test), except for the lysozyme- and the RNAzol only groups (Supplementary Figure 2). Of the non-kit-based RNA extraction methods, the lysozyme method produced the poorest quality but the highest yield of RNA.

**TABLE 1A:** RNA characteristics in column isolation-based group and organic isolation-based groups.

	Column isolation-based group {*n* = 8)
A260/280	2.07 ± 0.03
A260/230	2.13 ± 0.36
Standardized RNA yields (μg/g dry sample)	298 ± 136
RNA integrity number	5.63 ± 0.48

*RNA purity (A260/280; A260/230), standardized yields and RIN (RNA integrity number) for column isolation-based group.*

**TABLE 1B:** RNA purity (A260/280; A260/230) and standardized yields for organic isolation-based groups before additional purification step.

	RNAzol only (*n* = 8)	Beads-beating (*n* = 8	Lysozyme (*n* = 8)	Cell disruption (*n* = 8)	Lysozyme + beads{*n* = 8)
A260/280	1.60 ± 0.03	1.61 ± 0.04	1.63 ± 0.04	1.59 + 0.03	1.66 ± 0.04
A260/230	0.76 ± 0.06	0.81 ± 0.12	0.88 ± 0.11	0.82 ± 0.13	0.91 ± 0.12
Standardized RNA yields (μg/g dry sample)	283.20 ± 99.65	262.59 ± 51.32	355.59 ± 81.89	301.95 ± 168.25	307.41 ± 133.23

**TABLE 1C:** RNA purity (A260/280; A260/230), standardized yields and RIN (RNA integrity number) for organic isolation-based groups after additional purification step.

	RNAzol only (*n* = 4)	Beads-beating (*n* = 4)	Lysozyme (*n* = 4)	Cell disruption (*n* = 4)	Lysozyme + beads (*n* = 4)
A260/280	1.91 ± 0.07	1.93 ± 0.06	1.83 ± 0.26	1.97 ± 0.09	1.95 ± 0.05
A260/230	1.38 + 0.41	1.50 ± 0.30	1.59 ± 0.27	1.61 ± 0.36	1.60 ± 0.31
Standardized RNA yields (pg/g dry sample)	180.75 ± 101.77	177.26 ± 88.80	233.97 ± 126.74	200.70 ± 147.26	233.13 ± 130.48
RNA integrity number	2.675	2.675	2.65	2.55	2.575

*The variability reflects the standard deviation.*

### Determination of the Active Microbiome Associated With Freshwater Benthic Biofilms After Different Extraction Strategies

Meta-transcriptomic analysis was conducted to study the active microbiome, gene expression patterns and associated pathways in biofilm samples subjected to various RNA extraction strategies. HiSeq2500 analysis generated approximately 103, 92, 66, 96, 84, and 81 million paired-end raw reads from the samples subjected to the standard kit (K), RNAzol only (R), bead-beating (B), lysozyme (L), pressure bomb (P), and lysozyme + beads (LB) RNA extraction strategies, respectively. In all of the treated samples, the active microbiome generally comprised bacteria, viruses, eukaryotic microbes and archaea (Supplementary Figure 3). Notably, the RNA from samples subjected to kit extraction was characterized by a high relative abundance of active archaea and eukaryotic microbes and the lowest relative abundance of bacteria (S1). In contrast, all of the other RNA extraction methods were characterized by a higher relative abundance of active bacterial communities. Interestingly, the biofilms extracted using the pressure-based lysis method (P) had the highest relative abundance of viruses among all of the tested groups, while the samples subjected to the bead-beating lysis method (B) had the highest relative abundance of active eukaryotes.

**FIGURE 2:**
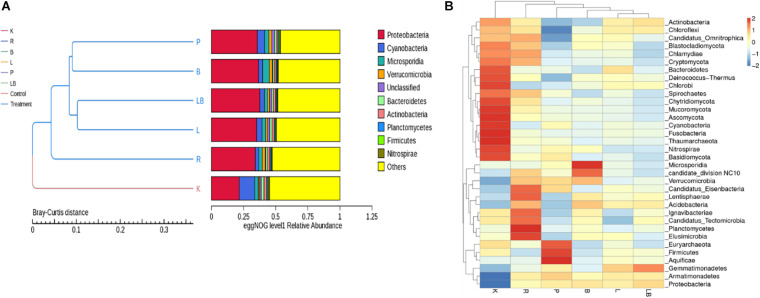
Analysis of the active microbiome in freshwater benthic biofilms subjected to different RNA extraction methods. **(A)** EggNOG level 1 relative abundance of biofilm communities at the gene expression level for each extraction group. **(B)** Heat map depicting the 35 most dominant phyla at the gene expression level. Abbreviations of extraction groups denote, K: Column-based kit isolation; R: RNAzol only group; B: Bead-beating group; L: Lysozyme group; P: Cell disruption bomb; LB: Lysozyme + Bead-beating group.

At the phylum level, Proteobacteria, Cyanobacteria, Actinobacteria, Verrucomicrobia, Bacteroidetes, Actinobacteria, Planctomycetes, Firmicutes, and Nitrospirae were most abundant in the combined column- and organic isolation-based groups (Figure 2A), followed by a fungal phylum (Microsporidia) and an unclassified phylum. The samples subjected to column isolation-based group produced a taxonomic cluster distinct from that of the other RNA extraction groups, which was attributed to the uniquely high relative abundance of active Cyanobacteria in the former group. The organic isolation-based groups clustered according to the type of lysis method, such that mechanical methods [pressure-based (P) and bead-beating (B)] clustered together, while enzymatic methods [lysozyme (L) or lysozyme + beads (LB)] formed a separate sub-group. The active microbiome diversity profile after RNAzol-based RNA extraction was found to be different between the column isolation-based and organic isolation-based groups.

A further comparison of inter-sample differences in the dominant 35 phyla (Figure 2B) revealed a noticeable difference in the microbial profiles. The samples subjected to kit RNA extraction (K) had higher relative abundances of Bacteroidetes, Deinococcus-Thermus, Chlorobi, Spirochetes, Chytridiomycota, Mucoromycota, Ascomycota, Cyanobacteria, Fusobacteria, Thaumarchaeota, Nitrospirae, and Basidiomycota than the samples extracted using other methods. However, the kit-extracted samples had the lowest relative abundances of Proteobacteria, Armatimonadetes, Verrucomicrobia, Candidatus_Tectomicrobia, Acidobacteria, and Gemmatimonadetes. The samples subjected to RNAzol RNA extraction (R) were characterized by the highest relative abundances of Candidatus_Eisenbacteria, Lentisphaerae, Ignavibacteriae, Candidatus_Tectomicrobia, Planctomycetes, and Elusimicrobia and exhibited the best representation of the 35 most dominant phyla. The samples subjected to pressure bomb lysis method (P) were characterized by the highest relative abundances of Euryarchaeota, Firmicutes, and Aquificae and the lowest relative abundance of Actinobacteria, Chloroflexi, and Candidatus_Omnitrophica, while those subjected to the bead-beating method (B) were characterized by the highest relative abundances of Microsporidia and Candidate_division NC10. The samples subjected to lysozyme (L) and lysozyme + bead-beating (LB) were clustered together and were constituted of an evenly distributed phyla as observed by their relative abundance profiles.

### Functional Profiling of Active Microbiomes

**FIGURE 3:**
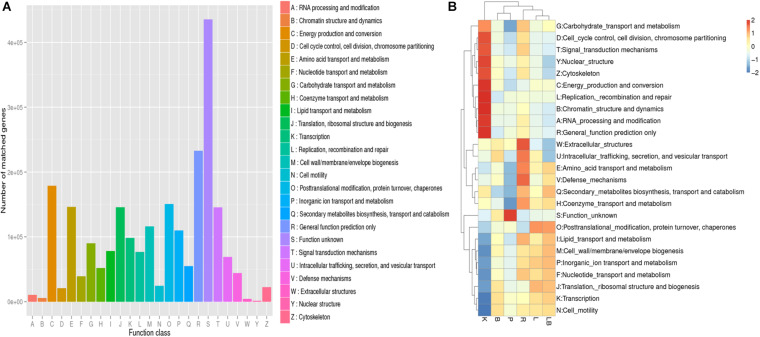
Non-supervised Orthologous Groups (EggNOG) annotation analysis of samples from freshwater benthic biofilms. **(A)** Numbers of genes matched to the EggNOG database in the combined column- and organic isolation-based groups. **(B)** Functional annotation analysis of the relative abundances in the six extraction groups, based on EggNOG database level 1 including 24 taxa. Abbreviations of extraction groups denote, K: Column-based kit isolation; R: RNAzol only group; B: Bead-beating group; L: Lysozyme group; P: Cell disruption bomb; LB: Lysozyme + Bead-beating group.

**FIGURE 4:**
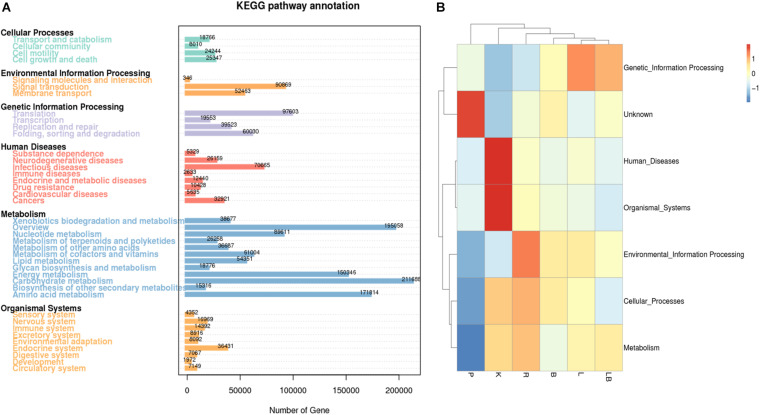
Kyoto Encyclopedia of Genes and Genomes (KEGG) pathway and functional annotation analysis of freshwater benthic biofilm samples. **(A)** Number of genes annotated to KEGG database at level 2 in the combined column- and organic isolation-based groups. *X*-axis indicates the number of genes annotated in each KEGG pathway; *Y*-axis indicates the name of KEGG level 2 pathways. **(B)** Functional annotation analysis of the relative abundances in the six extraction groups, based on KEGG database level I including 6 pathways. Abbreviations of extraction groups denote, K: Column-based kit isolation; R: RNAzol only group; B: Bead-beating group; L: Lysozyme group; P: Cell disruption bomb; LB: Lysozyme + Bead-beating group.

**FIGURE 5:**
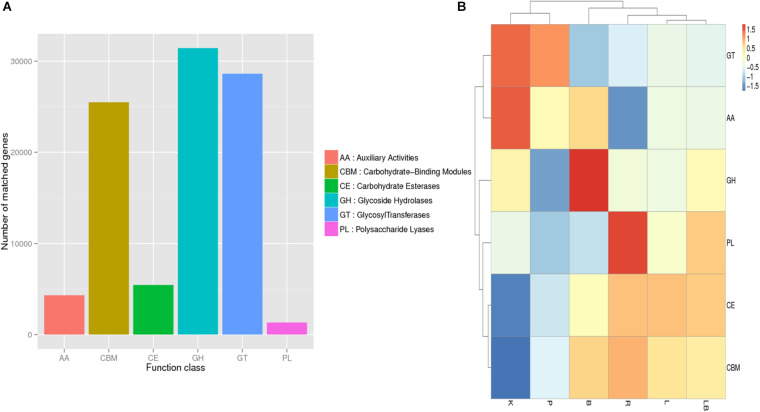
Carbohydrate-Active Enzymes Database (CAZy) annotation analysis of freshwater benthic freshwater biofilm samples. **(A)** Numbers of genes annotated to the CAZy database in the combined column- and organic isolation-based groups. **(B)** Functional annotation analysis of the relative abundances in the six extraction groups, based on CAZy database level 1 including six major function classes. Abbreviations of extraction groups denote, K: Column-based kit isolation; R: RNAzol only group; B: Bead-beating group; L: Lysozyme group; P: Cell disruption bomb; LB: Lysozyme + Bead-beating group.

To further our understanding of the effects of RNA extraction methods on the ability to investigate microbial metabolism in biofilm samples, the obtained transcripts were compared with the EggNOG (Figures 3A,B), KEGG (Figures 4A,B) and CAZy databases (Figures 5A,B).

All of the tested samples included genes involved in energy production and conversion, post-translational modification, amino acid and energy metabolism, translation, signal transduction, cell wall/membrane/envelope biogenesis, inorganic ion transport, and metabolism (Figure 3A). A comparison of the samples (Figure 3B) revealed that those subjected to the extraction kit protocol featured a distinct functional gene profile and the highest relative abundances of genes associated with carbohydrate metabolism; cell-cycle control; signal transduction; nuclear structure; cytoskeleton; energy production and conversion; replication, recombination and repair; chromatin structure and dynamics; RNA processing and modification; and general function. Interestingly, these samples also had the lowest relative abundances of genes associated with lipid transport and metabolism; cell wall biogenesis; inorganic ion transport and metabolism; nucleotide transport and metabolism; translation; transcription; and cell motility. By considering the active microbiome profile at the phylum-level (Figure 2A), the observed differences may have been attributed by a bias toward Cyanobacteria and Proteobacteria as a result of RNA extraction using the standardized kit method.

A comparison of the other RNA extraction methods and their impacts on functional gene annotation revealed that the samples subjected to mechanical lysis (namely bead-beating or pressure bomb) were clustered together and had similar functional gene annotation profiles. These samples were characterized by a generally lower relative abundance of annotated gene functions relative to the other samples. The samples subjected to pressure bomb lysis method contained the highest relative abundance of genes with unknown function. The samples subjected to lysozyme or lysozyme + bead extraction had similar annotated gene function profiles and were clustered together. The samples subjected to RNA extraction with RNAzol alone exhibited a distinct functional annotation pattern, with the highest relative abundance of genes associated with extracellular structures; intracellular trafficking; amino acid transport and metabolism, defense mechanisms, secondary metabolites biosynthesis, transport and catabolism; and coenzyme transport and metabolism.

Most of the genes represented in the KEGG pathway analysis were associated with metabolic pathways (Figure 4A). In particular, the category of carbohydrate metabolism was dominant, with 211,688 associated genes. In contrast, RNA extraction using a pressure-based lysis strategy resulted in the lowest relative abundance of genes associated with metabolic functions (Figure 4B). Compared with samples extracted using other strategies, the latter samples were also characterized by a relatively low relative abundance of genes associated with cellular processes, environmental information processing, organismal systems and human disease and a higher relative abundance of genes with unknown functions. Kit extraction produced samples with a high relative abundance of genes associated with human diseases and organismal system pathways. Again, the samples subjected to extraction with lysozyme or lysozyme + beads were clustered together and had the highest relative abundance of genes associated with genetic information processing pathways.

Overall, the highest relative abundances were observed for genes encoding carbohydrate-binding modules (CBM), glycoside hydrolases (GH), and glycosyltransferases (GT) (Figure 5A). However, a comparison of samples extracted using different methods (Figure 5B) revealed that those subjected to kit extraction had the highest relative abundances of genes encoding GT and axillary activities (AA) and the lowest relative abundances of genes encoding carbohydrate esterases (CE) and CBM. The samples subjected to pressurized extraction had a low relative abundance of genes encoding polysaccharide lyases (PL), GH, CBM, and CE, and a high relative abundance of genes encoding GT and AA. The samples subjected to bead-beating lysis method had the highest relative abundance of genes encoding GH when compared with other tested samples. The samples subjected to RNAzol extraction had the highest relative abundance of genes encoding PL. The samples extracted using lysozyme with or without bead-beating exhibited similar relative abundances of genes encoding carbohydrate-active enzymes and were clustered together.

### Differential Expressional Analysis of Active Microbiomes

For the differential gene expression analysis, the relative unigene expression levels were calculated as FPKM. Here, genes differentially expressed between libraries were identified using the criteria of a corrected padj < 0.05 and log2 fold change (challenge group/control group) ≥ 1. Differential gene cluster analysis was used to determine the patterns that emerged under different experimental conditions. Each challenge–control combination produced a differential gene set, and the FPKM values of all the combined differential gene sets in each experimental group/sample were included in the cluster analysis.

**FIGURE 6:**
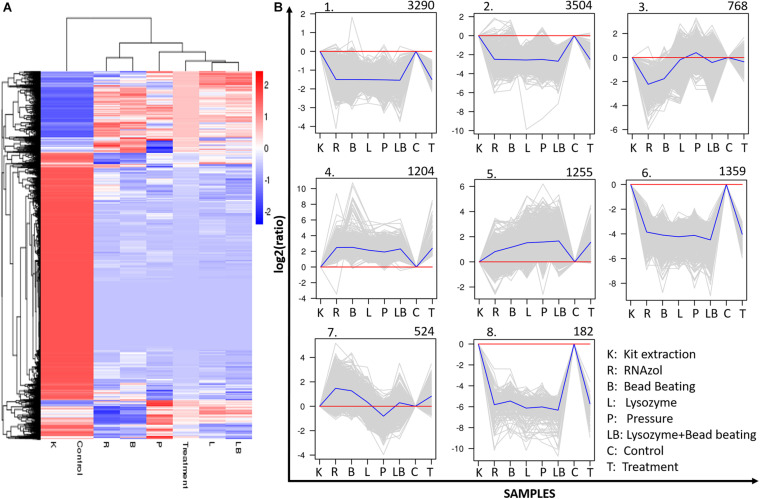
Clustering analysis of differentially expressed genes (DEGs). **(A)** Hierarchical clustering graph of the 12086 DEGs based on the average log_10_ (FPKM + 1) values of all genes in each cluster. Red bands indicate higher gene expression, while blue bands indicate lower gene expression. **(B)** Clustering of the 12086 DEGs into eight subclusters. The number of genes in each cluster is presented at the top of each cluster. Blue lines indicate the average relative expression levels in each subcluster relative to a log2 ratio of 0, which is represented by the red line. Gray lines represent the relative expression levels of each gene in each cluster. The sample names and corrected expression levels are presented on the *x* axis and *y* axis, respectively.

A total of 12,086 upregulated or downregulated DEGs (adjusted *p* value of < 0.05) were detected by comparing the gene expression levels in samples subjected to different RNA extraction strategies (Figure 6). A cluster analysis was used to determine the clustering patterns of DEGs under different experimental treatments according to the Euclidean distance method associated with complete linkage (Li et al., 2017; Figure 6A). The resulting clustering pattern suggested that the choice of RNA extraction method may influence the expressed gene profile in a freshwater benthic biofilms samples. The 12,086 identified DEGs were grouped into eight sub-clusters with various expression patterns (Figure 6B) after comparing the column- and organic isolation-based groups. Clusters 1 (3,290 genes), 2 (3,504 genes), 3 (768 genes), 6 (1,389 genes) and 8 (182 genes) contained downregulated genes, while clusters 4 (1,204 genes), 5 (1,255 genes), and 7 (524 genes) contained upregulated genes.

## Discussion

Typically, environmental freshwater benthic biofilms are comprised of a consortia of various organisms, as well as various impurities such as sand, silt and soils containing humic acid and fulvic acid (Wang et al., 2012). These impurities may contribute to the exogenous degradation of fragile RNA and thus decrease the integrity of this nucleic acid, especially when RNases are present. These challenges are commonly encountered during field-based biofilm studies, particularly when RNA isolation is required. High RNA integrity is essential for transcriptome analysis, as a lower level of integrity would result in the reduced recovery of full-length poly-A RNA and the biased construction of a cDNA library (Chen et al., 2014). A RIN ≥ 7.0 usually is considered acceptable for most RNA-seq protocols that ensures a low contamination of the small RNA fraction with degradation products (Jahn et al., 2008). For metatranscriptomic sequencing of benthic biofilms, we recommend not to use RNA sample with RIN ≤ 7 and to carry out an additional extraction. Consequently, in biofilms research, efforts have been made to use different RNA extraction methods for meta-transcriptomic sequencing, including the acid hot phenol method, MoBio PowerBiofilm RNA isolation kit (MO BIO Laboratories) and RNA Power soil kit (MO BIO Laboratories). However, the resulting levels of RNA integrity have not been explored (Schneider et al., 2015; Nakamura et al., 2016; Rampadarath et al., 2017).

To date, no standard sampling and processing methods for environmental biofilms have been developed. RNA degradation can be avoided by immediately subjecting environmental biofilm samples to extraction or by shock-freezing samples for storage at −80°C until extraction is possible. Raw biofilm samples can also be stored in DNA/RNA shield (Zymo Research, United States) or treated with RNAlater^TM^ solution (Invitrogen, United States) to prevent RNA degradation. McCarthy et al. (2015) analyzed the impact of different RNA isolation methods, as well as lysis and preservation methods on extracted RNA quality of acquatic microbial samples. Results revealed that the use of RNA preservation agents, such as RNAlater and RNAprotect (QIAGEN), not only benefited RNA integrity, but also prevented variations in expression profiles and therefore was recommended for transcriptomic studies. Therefore, the comparisons in our study provide a practical demonstration of the efficiencies of a selection of high-grade RNA isolation methods.

Our results demonstrated that total RNA isolation from freshwater benthic biofilm samples using a commercial kit yielded the best outcomes in terms of RNA purity and integrity. Moreover, RNA extraction using chemical reagents in the absence of an additional purification step yielded RNA with a low purity. The poor-quality outcomes of chemical reagent-based methods may be attributable to the impurities present in environmental samples. In contrast, the column based-kit isolation method was better suited to the removal of impurities such as humic acid and fulvic acid, which have been shown to affect RNA purity and integrity (Wang et al., 2012). Although the inclusion of an additional purification step after chemical extraction improved RNA quality, the integrity of RNA in those samples remained at impracticable levels. Our results emphasize that the RNA isolation method must be selected carefully, as the combination of RNA purity, yield and integrity number (RIN) determines the success of downstream molecular biological applications such as next-generation sequencing, qRT-PCR and microarrays (Fleige and Pfaffl, 2006). In a previous study, various RNA extraction methods ranging from commercially available kits and TRIzol to the phenol/isopropanol method were applied to a biofilm-producing microbial strain: *Staphylococcus aureus*. In that study, gel electrophoresis revealed clear differences in the integrity of the extracted RNA. Although that study investigated only a single-species biofilm, the authors similarly concluded that the choice of RNA extraction method affected the RNA integrity (Atshan et al., 2012). Franca et al. (2011) have compared three RNA extraction commercial kits on biofilm samples of *Staphylococcus epidermidis*, and the results revealed clear differences in the quantity and purity of extracted RNA. Real-time quantitative PCR analysis showed that the normalized expression of a well-known virulence gene of *S. epidermidis*, *icaA* that RNA extracted with PureLink^TM^ was significantly lower than the other kits tested. The take-home message from the above-mentioned studies is that researchers need to take special care when selecting RNA extraction kits and cDNA synthesis kits for their investigations, as the choice may significantly affect the downstream RNA-based analysis. While most RNA-based biofilm studies focus on monospecies models, more studies should consider to use RNA-based investigations on environmental multispecies biofilms.

Our exploration of cell-disruptive methods, including RNAzol, failed to produce any significant improvement in the RIN. In other words, the mechanical, chemical and enzymatic-based disruption methods tested in this study led to similar RNA integrity, purity and yield levels. This suggested that the poor RIN problem of chemical isolation-based groups may not be associated with lysis procedures, but with the RNAzol method itself. In addition, this suggested that besides the choice of RNA isolation method, sample collection and storage may constitute an essential step, as the RIN of column-based kit extraction is not very good either (RIN = 5.4). Thus, it is recommended to apply some RNA preservative agents during sample collection.

The significant difference in the active microbiomes between the column- and organic-based isolation was foreseeable, implied by the huge difference observed in RNA integrity levels between the two major procedures. Considering the unqualified RIN performance observed in both column isolation-based group and chemical isolation-based groups in this study, future studies should optimize the sample collection and storage method prior to RNA extraction to be able to yield RNA with acceptable integrity levels that can be proceed with downstream sequencing and analysis. More specifically, it is advisable to use RNA preservatives. It has been shown that compared to the samples without preservative substances, the use of preservatives significantly increased RIN from 2.7 to 8.7 (McCarthy et al., 2015). However, the results observed in this study showing significant discrepancy in the active microbiomes among groups still provided valuable insights for future perspectives. The column isolation-based group was characterized by the highest relative abundances of Archaea and Eukaryota, whereas the organic isolation-based groups generally had the highest relative abundances of bacteria. The extremely low relative abundance of active bacteria in the column isolation-based group samples indicates that researchers exploring the bacterial microbiome should avoid using kits for RNA isolation. Instead, column-based procedure might be superior for omics studies of the Archaea or Eukaryota microbiomes. RNAzol group was characterized by the high relative abundances of Bacteria, with extremely low abundance of Viruses and relatively low relative abundance of Archaea and Eukaryota, indicating that researchers may consider using this method when their investigations are mainly focusing on Bacteria. Beads-beating group was characterized by the high relative abundance of Eukaryota, with averaged relative abundance of Bacteria and low relative abundance of Archaea and Viruses, suggesting that this method would be appropriate for studies of Eukaryota microbiomes. As for the pressure bomb method, the extremely high relative abundance of Viruses indicated that researchers investigating viral microbiome could consider this method as a most appropriate candidate. The high relative abundance of Viruses and Bacteria presented in Lysozyme group suggested this method might be a good option for omics studies of Bacteria and Viral microbiomes.

Most importantly, the lysozyme + beads group demonstrated the best outcomes showing highest relative abundance of Bacteria among all groups, suggesting that a combination of chemical, mechanical and enzymatic lysis procedure had the best performance in breaking down bacteria cell walls and released most abundant bacteria cells. Considering that the bacterial cell walls are significantly thick in Gram-positive bacteria as it is mainly composed of peptidoglycan (Salton and Kim, 1996), insufficient cell lysis may not effectively breakdown these cells, consequently, yielding lower amounts of RNA that can affect downstream applications such as metatranscriptome sequencing and analysis. For omic studies focused on bacteria microbiome of environmental biofilms, researchers should incorporate varied cell lysis strategies to ensure an efficient cell lysis outcome.

Notably, we observed clear differences in the functional profiles between the column- and organic isolation-based groups. The column isolation-based group functional profile exhibited the highest relative abundance of genes associated with human diseases and organismal systems, and the lowest relative abundance of genes associated with metabolism. This pattern is attributable to the high relative abundance of Eukaryota in the column isolation-based group. In the organic isolation-based groups, KEGG annotation analysis revealed high relative abundances in the categories of cell growth and death, transcription, translation and metabolism of amino acid and secondary metabolites (Supplementary Figure 4). We hypothesize that the significant differences in gene expression are most likely attributed to the use of different RNA extraction methods. It is well known that the RNA distribution within cells is not uniform. Despite the widespread acknowledgment that mRNAs are differentially localized within the cell, few studies have examined whether “common” RNA extraction methods are equivalent in their abilities to extract differentially localized RNA species, and whether the method of RNA isolation affects our ability to detect differentially expressed transcripts. Sultan and colleagues compared two RNA isolation methods on HEK_293_ human cells (Qiagen RNeasy kit and guanidinium-phenol (TRIzol) extraction) and two library selection schemes (poly-A enrichment and rRNA depletion) on downstream transcript abundance estimates, and found that rRNA depletion was particularly sensitive to the RNA extraction method (Sultan et al., 2014). In another study by Franca, a similar conclusion has been drawn in comparing gene expression profiles following RNA extractions using various kits on *S. epidermidis* biofilm samples (Franca et al., 2011).

The superior efficiency of kit-based RNA extraction relative to RNAzol-based extraction is expected. However, the results of this study emphasize the need for careful consideration when selecting a method for RNA extraction from freshwater biofilm samples. The selected method may significantly affect the downstream NGS sequencing results, and the potential biases related to the choice of extraction method may include shifts in the active microbiome and functional attributes.

The findings of this study have to be seen in light of some limitations. This study selected 16S rRNA gene sequencing to identify the microbial community structure and metatranscriptomics analysis to reveal the RNA-based community structure and functional attributes. Unfortunately, this study did not conduct a whole-genome metagenomic sequencing, and hence, the comparison between functional potentials and functional activities in this study, was therefore not possible. It would, therefore, be advisable to involve whole genome sequencing result as a basis for metatranscriptome analysis, in similar future studies. Additionally, the application of novel technology such as acoustic energy, capable of rapid, unbiased and efficacious disruption of cellular membranes, without the use of chemicals or enzymes (Branch et al., 2017), can be considered as an ideal alternative to cell disruption method before DNA/RNA extractions. However, more studies should be performed to verify the effectiveness of the acoustic method on biofilm samples.

## Conclusion

Our study revealed that different RNA extraction methods not only produce varying levels of RNA quality and yield but may also significantly affect the results of downstream applications such as metatranscriptomics sequencing and analysis. Specifically, the choice of extraction method may lead to a shift in the observed active microbiome and outcomes of functional analyses. Our findings provide caveats for researchers when performing RNA extractions, as the RNA isolation method should be carefully selected and optimized according to their specified study purposes.

## Author’s Note

Our findings point to significant discrepancies of the active community structure profiles resulting from the various RNA extraction strategies, with some methods being suited for studying eukaryotic organisms, while others for bacteria. The results reported in this study suggest the importance of carefully considering the choice of RNA extraction method on freshwater biofilm samples, as this may strongly affect the downstream NGS sequencing results. The biases of using one extraction method from another include shifts in active microbiomes and functional attributes.

## Data Availability Statement

The datasets presented in this study can be found in online repositories. The names of the repository/repositories and accession number(s) can be found below: National Center for Biotechnology Information (NCBI), https://www.ncbi.nlm.nih.gov/, PRJNA594486.

## Author Contributions

YY collected the data. YY and OH conceived the conception or design of the work. YY and OH drafted the manuscript. YY, SR, and OH revised the manuscript. All authors analyzed and interpreted the data.

## Conflict of Interest

The authors declare that the research was conducted in the absence of any commercial or financial relationships that could be construed as a potential conflict of interest.
