# Supplementary material for: Active Microbiome Structure and Functional Analyses of Freshwater Benthic Biofilm Samples Influenced by RNA Extraction Methods
Source: Front Microbiol. 2021 Apr 16;12:588025. doi: 10.3389/fmicb.2021.588025 (PMC8085529; doi:10.3389/fmicb.2021.588025)
Supplement: Supplementary file 1 [file Data_Sheet_1.docx]

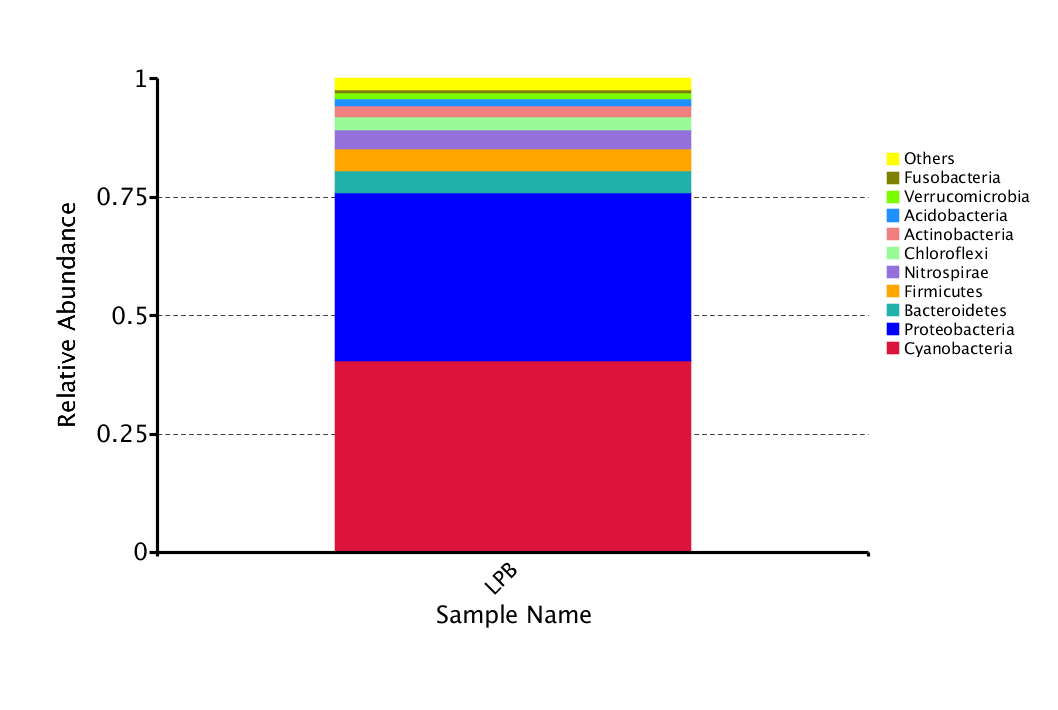


Supplementary Figure 1: Relative abundance of 16S rRNA reads of the Lily Pond Biofilm (LPB) sample at the phylum level.


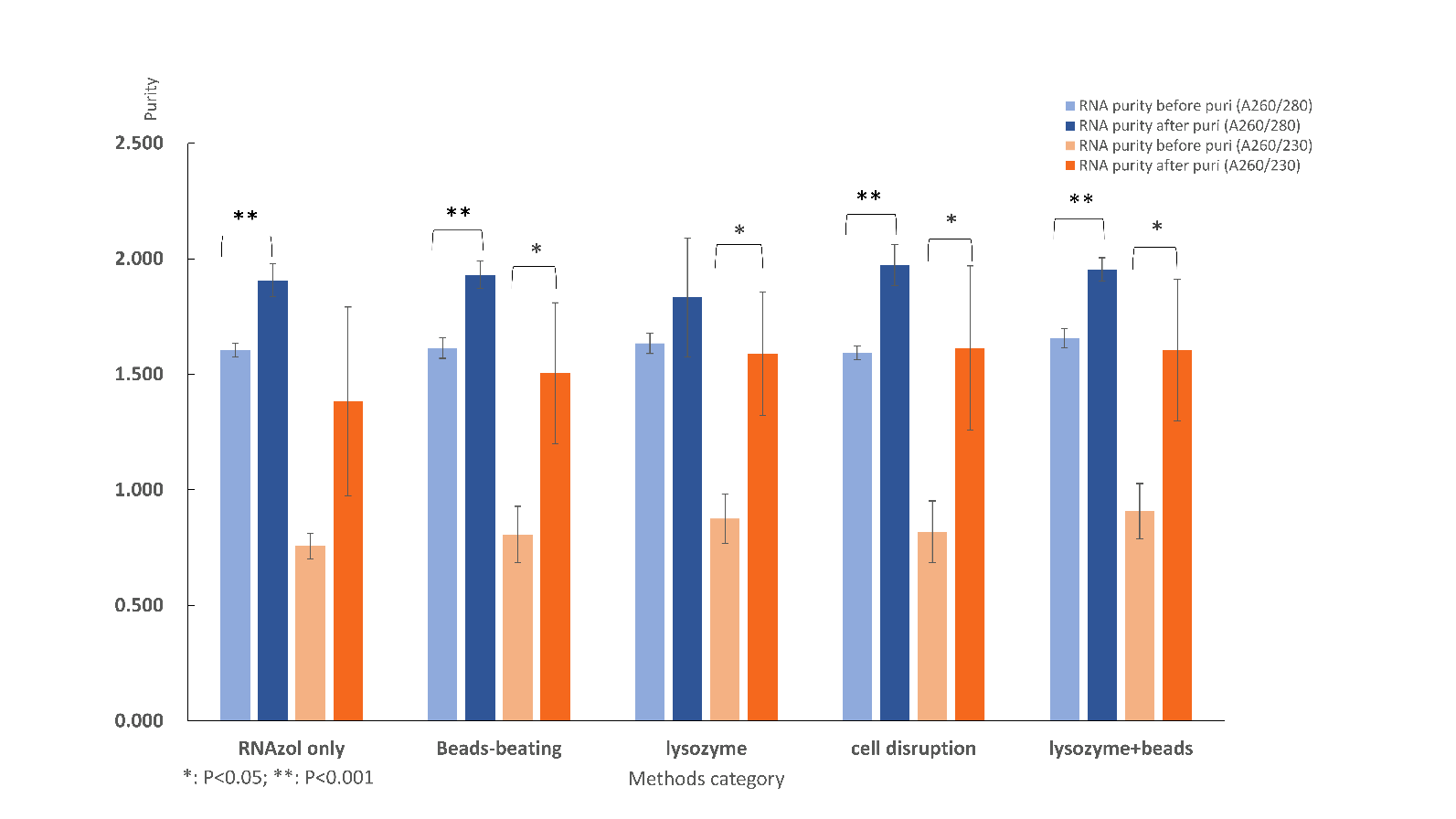


Supplementary Figure 2: comparison of RNA purity (A260/280; A260/230) for five RNAzol-based extraction groups before and after post-isolation purification step.


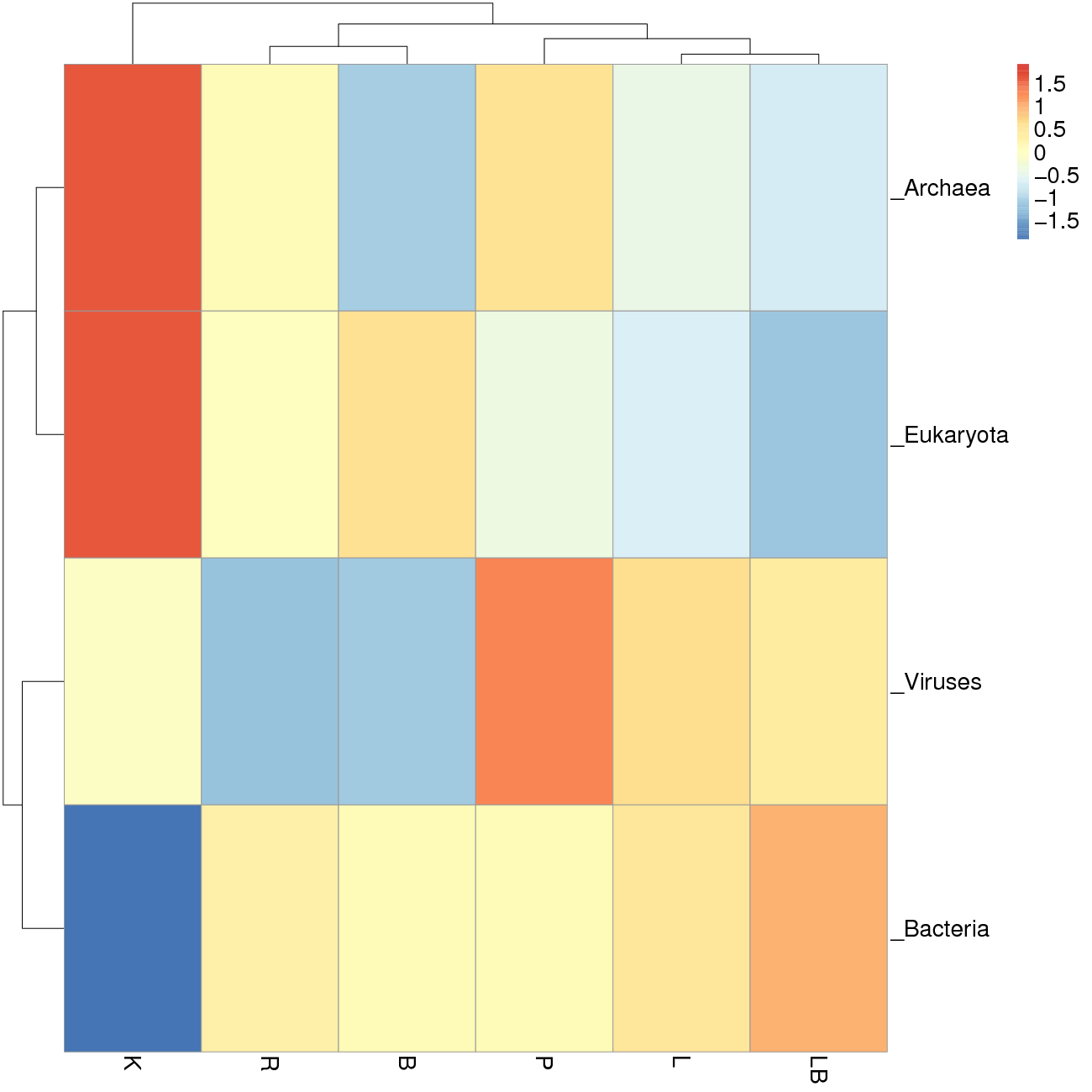


Supplementary Figure 3: Kingdom-level species annotation derived from taxonomically annotated reads of the six RNA extraction groups. The color code denotes a) For highest value of relative abundance <red> , b) For lowest value of relative abundance <blue>. Abbreviations of extraction groups denote, K: Column-based kit isolation; R: RNAzol only group; B: Bead-beating group; L: Lysozyme group; P: Cell disruption bomb; LB: Lysozyme + bead-beating group.


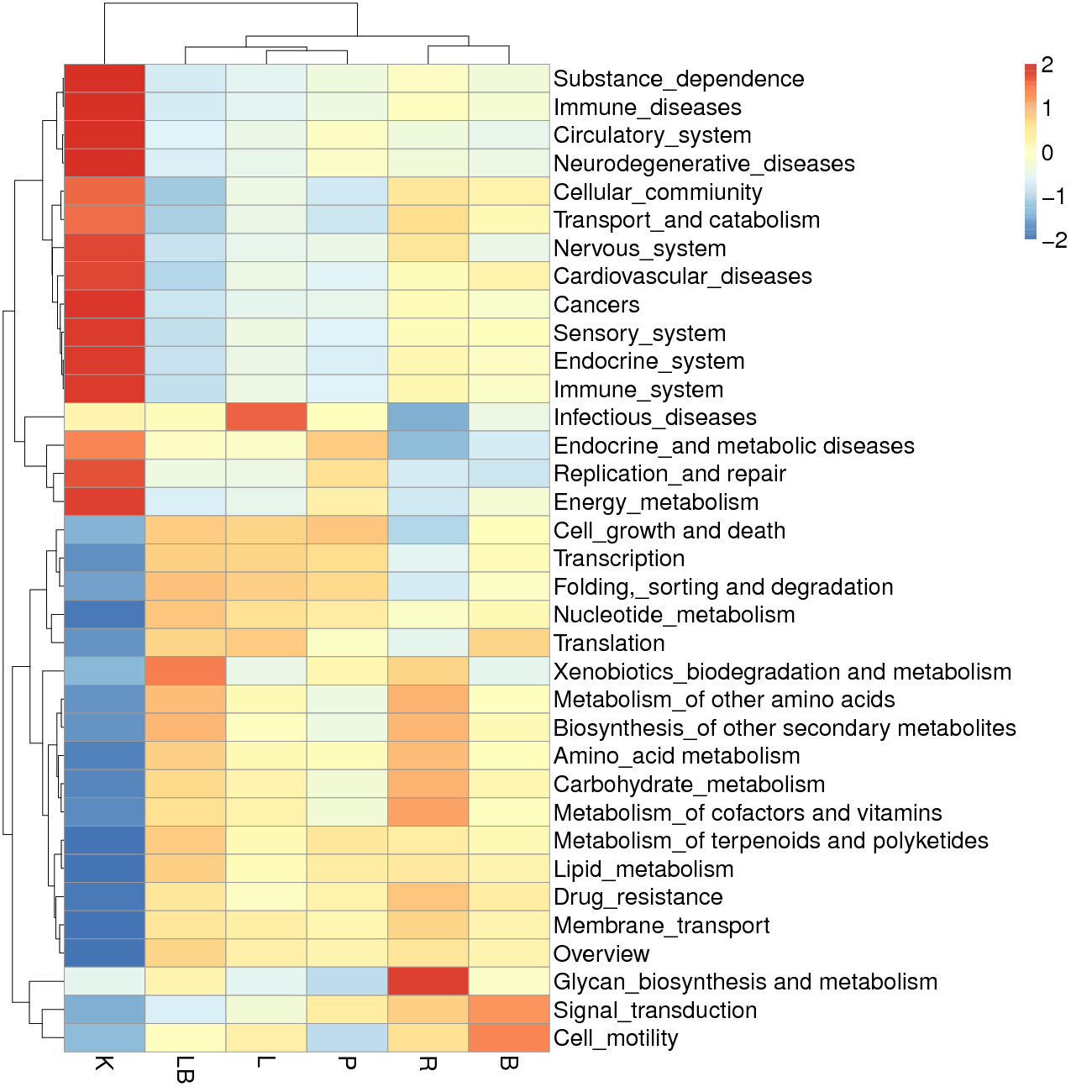


Supplementary Figure 4: KEGG pathways derived from functionally annotated genes following six RNA extraction groups based on KEGG database level 2 - 43 sub-pathway database. The color code denotes a. For highest value of relative abundance <red> , b) For lowest value of relative abundance <blue>. Abbreviations of extraction groups denote, K: Column-based kit isolation; R: RNAzol only group; B: Bead-beating group; L: Lysozyme group; P: Cell disruption bomb; LB: Lysozyme + bead-beating group.


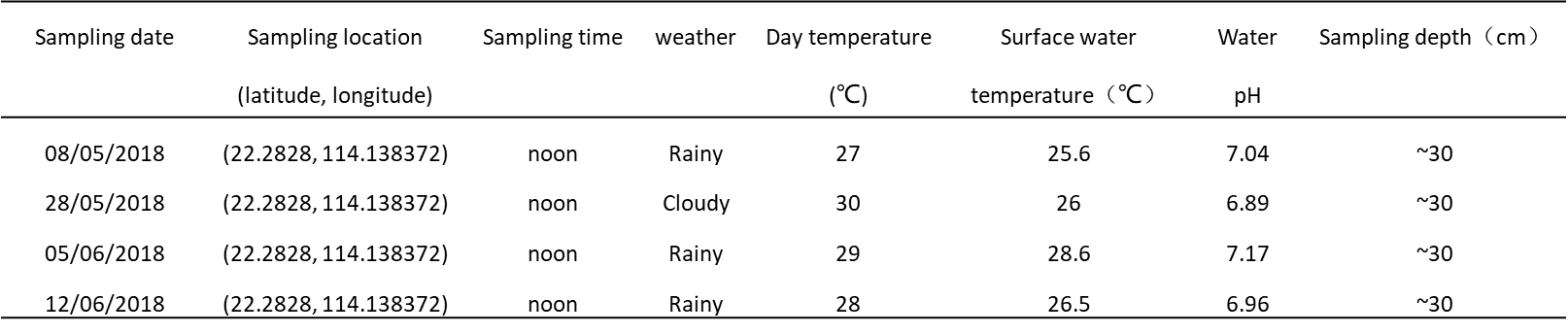
Supplementary Table 1: Lily pond sampling metadata.


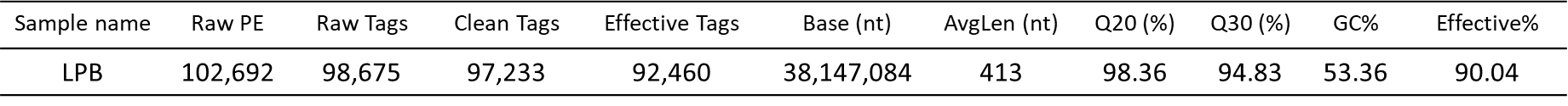


Supplementary Table 2: Preprocessing of 16s rRNA gene sequencing data and QC statistics.
